# Quantum technologies and geopolitics: comparing parliamentary rhetoric

**DOI:** 10.1140/epjqt/s40507-025-00456-w

**Published:** 2025-12-19

**Authors:** Viktor Suter, Gina Pöhlmann, Charles Ma, Miriam Meckel

**Affiliations:** School of Management, Economics, Law, Social Sciences, International Affairs and Computer Science, University of St. Gallen, St. Gallen, Switzerland

**Keywords:** Quantum technologies, Dual-use technology, International relations, National security, Parliamentary speech

## Abstract

Quantum technologies are rapidly emerging as a strategic priority for global political powers. Yet little is known about how policymakers across countries perceive the security implications of quantum technologies, even though such perceptions shape policy priorities and public understanding of technological threats. Drawing on securitization theory, we analyze parliamentary speeches from 2010 to 2024 across Australia, the United Kingdom, the United States, the European Union, and Singapore. Using computational social science tools, including a novel method based on large language models to quantify security emphasis, we report on three principal findings. First, attention and security framing vary markedly across legislatures, with the United States showing the highest intensity, the United Kingdom a moderate pattern, and Australia comparatively muted security rhetoric despite frequent discussion. Second, security emphasis rises over time in every parliament studied. Third, highly securitized debates cluster around the topics of transitions to quantum-secure communication infrastructures, great-power competition and alliances (including AUKUS), and the regulation of cross-border capital, knowledge, and technology flows. The study contributes cross-national, longitudinal evidence on how quantum technologies are politicized.

## Introduction

Quantum sensing, computing, and communication, collectively called quantum technologies, are attracting heavy public and private investment. Quantum sensing is the most mature of these technologies, with commercial applications already available and successful deployment in fields like medical imaging. Quantum communication is being field-tested for ultra-secure data transmission, while quantum computing remains the most experimental and challenging, with researchers still building prototypes and debating fundamental scalability questions [15]. Yet enthusiasm over the economic opportunities and scientific advancements is tempered by concerns about the impact of quantum technologies on international security [37].

Previous studies have shown that quantum technologies play a central role in matters of technological sovereignty, geopolitical rivalry, and military applications [19]. Global actors such as NATO and the European Commission treat quantum technologies as strategic assets because they offer hard-to-break cryptographic capabilities [12]. These benefits fuel protectionist impulses, such as export controls and restricted collaborations, which, if pursued too aggressively, risk sliding into isolationism. At the same time, many Latin American and African states struggle to build quantum technology capabilities because of limited resources and competing policy priorities [40]. This kind of uneven progress may accentuate the power imbalances between nations.

In addition, the governance of quantum technologies develops in specific national legal and political contexts, where differing regulatory traditions shape outcomes concerning fundamental rights, security priorities, and the application of precautionary principles. These distinct innovation systems manifest in divergent national strategies: the United States emphasize market-driven *“permissionless innovation”*, the European Union prioritizes a values-based orientation, and China pursues a state-led, top-down approach [18]. Amid these varied national frameworks, international standardization efforts, particularly in Europe, seek to harmonize technical specifications and facilitate interoperability across different governance models [36]. Recent scholarship illustrates ongoing debates about these governance priorities, with some arguing that national frameworks insufficiently address ethical concerns relative to expert recommendations [1]. These contrasting philosophies manifest in how governments respond to emerging quantum technologies. Cross-national comparison of parliamentary discourse is therefore essential for understanding how different strategic cultures affect threat perceptions and policy priorities.

Although scholars have mapped the political implications of quantum technologies, we lack a cross-country, multi-year account of how elected officials perceive the security issues surrounding them. Parliamentary speeches are particularly valuable in this regard because they capture the priorities and framing choices of elected officials. While governmental and policy reports provide valuable insights into official strategies [29, 38], speeches offer a distinct perspective on how quantum technologies are politically constructed. Policy reports are typically the end product of lengthy negotiations and represent consolidated government positions. In contrast, parliamentary speeches capture policymaking in progress, i.e., how elected representatives frame issues for their constituents, engage in contestation, and build consensus around particular narratives. Importantly, speeches provide a platform for voices that may be absent from official reports, exposing the range of security framings that compete in parliamentary deliberation.

Our analysis addresses this gap. We analyze a corpus of 935 relevant paragraphs extracted from speeches delivered from 2010 to 2024 in the parliaments of Australia, Singapore, the United Kingdom, the United States, and the European Union. These cases were selected to capture variation in governance approaches to emerging technologies. The United States and United Kingdom represent permissionless innovation models with strong military-technology complexes, while the EU prioritizes values-based regulation [18]. Australia and the UK, alongside the US, form the AUKUS alliance with explicit security cooperation commitments. Singapore, in contrast, offers a distinct democratic-authoritarian hybrid model with state-led technology development. This variation allows us to examine how different political systems and contexts affect the securitization of quantum technologies. Specifically, we ask:

**RQ1.**: How often do members of parliament in the sample countries frame quantum technologies as security issues, and how has this changed from 2010–2024?
**RQ2.**: What security-related topics emerge in parliamentary debates, and how do they differ across countries?

In addressing these research questions, our study makes two main contributions. First, we develop a measure of latent security emphasis by using large language models (LLMs) to rate parliamentary speeches on a scale from 0 to 100. We demonstrate the validity and scalability of this approach by showing that the resulting scores closely align with those from independent human coding. Second, we apply this measure to a longitudinal, cross-country dataset to examine how elected officials communicate about quantum technologies in relation to security issues. In doing so, we complement the existing social science literature on quantum technologies, which is predominantly conceptual and qualitative, with quantitative evidence.

The article proceeds as follows. Section 2 introduces securitization theory and positions quantum technologies within this literature. Section 3 details the data collection, processing, and validation pipeline. This includes the LLM-based text scaling task, as well as the topic modeling and sentiment analysis procedures. Section 4 presents the results and Sect. 5 concludes by discussing the study’s implications and limitations.

## Securitization theory and quantum technologies

According to Wæver [39], security issues are developments that threaten the independence of a state and its political order in a particularly dramatic fashion. Securitization theory, which developed in the disciplines of international relations and security studies, seeks to explain how political issues are turned into security issues [4]. The theory emphasizes the role of rhetoric, or “speech acts,” in constructing security issues. It posits that a political issue becomes a security issue not solely due to its objective danger but when influential actors (e.g., political leaders) declare it to be so [2]. This process of “securitization” often creates a pretext for action, such as emergency policies or military action, that go beyond politics as usual. Initially applied to issues like terrorism and migration, securitization theory has since been extended to study technology.

In the realm of technology and cybersecurity, Hansen and Nissenbaum [14] highlight that labeling digital technologies as national security concerns alters how they are perceived and managed. Although the primary focus often lies in the military and defense sectors, other domains of society (ranging from individual privacy and cultural identity to economic stability) may also be seen as vulnerable to cyber threats. A frequent feature of the securitization of digital technologies is the articulation of hypothetical disaster scenarios, which portray cyber incidents as producing cascading effects across critical infrastructures, potentially exposing the state and society to external control or disruption.

Further exploring the implications of securitization, Lacy and Prince [20], citing the 2015 Ukraine power grid hack and the 2016 Bangladesh Bank cyber heist, argue that while cyber threats are undeniably real, they are often framed through oversimplified threat narratives. Rather than treating cybersecurity as a uniform phenomenon, the authors [20] stress the importance of examining cybersecurity in specific contexts to better understand its complexity. In practice, this context-specific approach reveals a series of trade-offs, such as balancing infrastructure protection with openness to innovation in research, commerce, and international exchange, or weighing a state’s surveillance and monitoring powers against civil liberties [7]. As digital security challenges evolve, policymakers must navigate these competing interests in a constantly shifting landscape.

### Quantum technologies as an emerging security concern

Quantum technologies are undergoing securitization as states increasingly view them as security issues. Their dual-use potential, the capacity to serve both civilian and defense purposes, combined with intensifying global competition for technological superiority, has heightened security concerns. Recent policy analyses find that quantum is shifting from a generic emerging technology to a securitized, dual-use capability shaping export controls, research screening, and critical-infrastructure policy [27]. The literature identifies several specific threats:

- **Cryptographic Threat of Quantum Computing:** A scalable, fault-tolerant quantum computer could pose a fundamental threat to current cryptographic systems. While such a device does not yet exist, it could break widely used public-key encryption algorithms, compromise secure communications, and expose sensitive data across networks. This vulnerability affects all sectors (government, industry, and civil society) as virtually all digital security relies on these cryptographic foundations [12]. In response, the EU, United States, and other nations are proactively developing quantum-resistant solutions to mitigate these risks. These include post-quantum cryptographic standards and quantum communication infrastructures such as the European Quantum Communication Infrastructure [8].
- **Store Now, Decrypt Later:** A near-term risk is that adversaries collect sensitive encrypted data today and store it for future decryption when fault-tolerant quantum computers become viable. This poses a threat to confidential information, including government communications, trade secrets, and personal data [25].
- **Military and Defense Applications:** Adversaries gaining a decisive advantage in quantum technologies could fundamentally alter the military balance. Major powers, including the United States and China, are investing in the development of quantum technologies for defense applications. These include: GNSS-independent navigation systems;^1^ quantum-secured satellite and terrestrial communications; quantum sensing for submarine detection and underground mapping; quantum LiDAR^2^ and radar systems; and quantum computing to accelerate battlefield decision-making. Furthermore, quantum communication, particularly quantum key distribution, promises to protect military communications against both threats from current and future quantum-enabled espionage [15, 19].
- **Supply Chain Vulnerabilities:** Over-reliance on external actors for quantum technologies creates strategic vulnerabilities. Key dependencies include rare earth materials, ultra-precise manufacturing capabilities, specialized components (such as cryogenic systems and photonic devices), and proprietary software stacks. The EU’s Quantum Europe Strategy, for instance, identifies supply chain independence as a strategic goal [9].

Research is beginning to take security aspects like these into account. For example, a recent analysis of language in Canadian strategy documents shows that they adopted a more threat-focused tone, departing from earlier language that focused more on economic opportunity and scientific progress [24]. This shift is consistent with what some describe as a hype about quantum technologies [34], where threat narratives amplify dangers to national security. Similarly, security concerns underpin the strategies of allied governments (including Canada, the U.S., the European Union, New Zealand, and the UK) which prioritize post-quantum cryptographic standards, expanded defense cooperation, and the protection of critical ICT infrastructure [5].

Reports by policy research bodies and government agencies support these insights. For example, a report by the United Nations Institute for Disarmament Research states that quantum computing could both facilitate arms-control verification and undermine existing cryptographic safeguards, urging new international norms [23]. An FBI report similarly calls quantum R&D theft an immediate concern and cites efforts by “bad actors”, primarily China, to steal quantum know-how and innovations [10]. These developments suggest that quantum technologies are increasingly perceived in terms of security imperatives by both academics and policy experts.

In sum, securitization theory explains how political actors transform technological issues into matters of national security. Recent academic and governmental debate suggests that quantum technologies are entering this securitized space, framed increasingly in terms of espionage, infrastructure vulnerability, and geopolitical rivalry. Despite this shift, we lack empirical evidence of how elected officials themselves portray quantum technologies: Do they adopt security narratives? If so, how do they vary across countries and over time? Our study addresses that gap by providing the first explorative, longitudinal analysis of parliamentary debates on quantum technologies across five legislatures.

## Data & methods

We adopt a computational social science approach to text analysis. First, we use LLMs to quantify the latent security emphasis in texts. Known as text scaling and well-established in political science, this approach converts qualitative textual content into quantitative measures [3]. We take a novel approach, however, by relying on the reasoning and interpretation capabilities of modern LLMs rather than supervised or unsupervised classifiers [21]. Second, we apply BERTopic, a topic modeling technique that uses transformer-based embeddings and clustering [13], to identify topics in the data. Third, we apply an LLM-based sentiment analysis. Together, these methods allow for a data-driven exploration of the security-related rhetoric about quantum technologies. Below, we detail the case selection, data collection, pre-processing, analysis, and validation steps.

### Case selection

The selection of the five legislatures (the Australian Parliament, the UK Parliament, the US Congress, the European Parliament, and the Parliament of Singapore) follows an exploratory comparative design. The primary goal is not to make generalizable causal claims about specific types of political systems, but rather to map the landscape of parliamentary rhetoric and generate initial insights into how it varies across different contexts.

Cases were selected based on two considerations. First, all cases were required to meet essential inclusion criteria: (1) demonstrated policy engagement with quantum technologies in the form of national strategies, funding, or regulation; and (2) maintenance of accessible, verbatim parliamentary records suitable for systematic data collection. Second, the final selection was made to ensure variation across dimensions of technology governance and geopolitical posture. The United States and the United Kingdom were chosen as exemplars of the “permissionless innovation” model, characterized by market-driven development and strong military-industrial complexes [18]. The European Union provides a crucial contrast, representing a supranational entity that prioritizes values-based technology regulation [31]. Australia was selected for its AUKUS membership and explicit commitments to security cooperation on advanced technologies. Finally, Singapore represents a state outside Western military alliances with a hybrid democratic-authoritarian system and strong state-led approach to technology development. This variation enables comparison of how different governance models and strategic postures shape the securitization rhetoric.

### Data collection & pre-processing

The dataset consists of verbatim transcripts of parliamentary speeches. Data were collected from official records, including Australian Hansard,^3^ European Parliament Plenary Service,^4^ Singapore Hansard,^5^ UK Hansard,^6^ and US Congressional Record.^7^ The respective websites were queried for the term “quantum” in all 24 official EU languages for the European Parliament and in English for Australia, Singapore (where speeches originally delivered in Mandarin, Tamil, and Malay are translated and recorded in English), the UK, and the USA. The resulting metadata were downloaded via official interfaces or, where this was not available, scraped using the Beautiful Soup and Selenium Python packages. The metadata was filtered to the period from January 2010 to October 2024 and deduplicated before the relevant documents were downloaded and converted to text files.

To ensure consistency across the text corpus, all non-English text was translated to English using the DeepL API. For languages not supported by DeepL, such as rare instances of Irish and Maltese in the EU documents, Google Translate’s web interface was employed. Both tools have proven to deliver good quality translations for research purposes [6, 28, 30]. The resulting files were reviewed for relevance. Media articles, constituent letters, bills, funding tables, and other non-verbatim content included in the records were removed. Finally, paragraphs containing the term “quantum” were extracted. By manually reviewing the data, instances where “quantum” did not refer to quantum technologies, such as uses of the word denoting “quantity” or “amount”, were removed. This approach yielded 935 paragraphs, distributed as follows: 326 from the US, 297 from Australia, 215 from the UK, 58 from the EU, and 39 from Singapore. These paragraphs were subsequently used for text scaling tasks, topic modeling, and sentiment analysis.

### Text scaling

We quantified the security emphasis with an LLM-based “asking-and-averaging” procedure [21]. For every paragraph ($n=935$) we queried three LLMs (claude_sonnet_4_20250514, mistral_medium_2505, and gpt_4o_2024_08_06), using a fixed temperature of 0 to obtain outputs.^8^ Each model received the following prompt: 
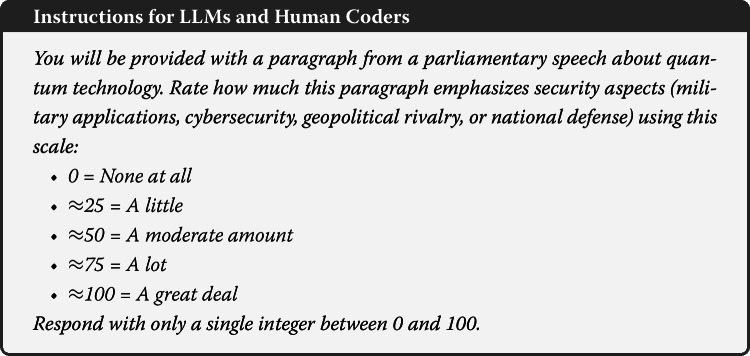
 The models produced one value per paragraph, which we averaged to yield one security score per paragraph.

To validate the LLM-generated scores, three human coders independently rated a random sample of $n=100$ paragraphs (roughly 10% of the text corpus). Agreement among human coders was high (Table 2; $\mathrm{ICC}=0.81$, $\alpha=0.80$), as was agreement among the LLMs that rated the same sample (Table 3; $\mathrm{ICC}=0.84$, $\alpha=0.79$). To assess human–LLM alignment, we averaged the scores within each group. The resulting ratings showed strong agreement (Table 4; $\rho =0.92$, $\mathrm{ICC}=0.90$, $\alpha=0.88$), indicating that the automated procedure closely approximates human judgment. Full reliability tables and visualizations are provided in Appendix A and Fig. 6.

### Sentiment analysis

To evaluate the tone of parliamentary discourse, we conducted a sentiment analysis using LLMs. The 935 paragraphs were evaluated by the three aforementioned LLMs (Sect. 3.3). The models received a standardized prompt that instructed them to classify the sentiment as positive, neutral, or negative based on the text’s emotional connotation: 
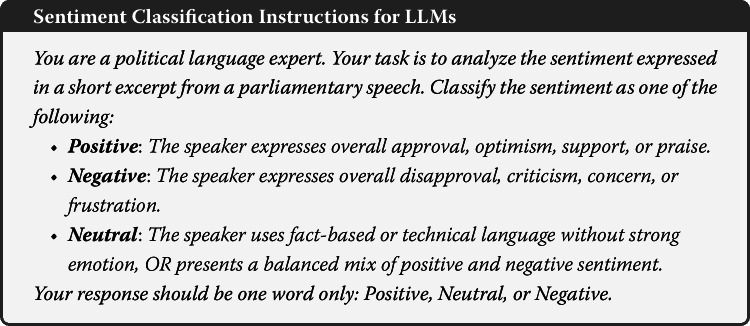
 Each model, set to temperature = 0, generated one sentiment label for every paragraph. A majority-vote rule was then applied to determine the final sentiment classification. In the rare cases where there was a tie (6 out of 935 paragraphs, or approximately 0.6%), the sentiment was set to neutral. As a robustness check, we applied a pre-trained RoBERTa sentiment classifier [22] to the dataset. As further explained in the Results section and in Appendix B, the outputs of this check align with the findings of the LLM-based approach to sentiment classification.

### Topic modeling with BERTopic

To identify the topics characteristic of high-security rhetoric, we applied BERTopic to the subset of paragraphs with security scores above 66 points ($n=201$). Prior to modeling, we preprocessed the text by removing stop words and common quantum-related phrases (e.g., “quantum computer”), which appear throughout the corpus and offer limited discriminatory value. Text embeddings were generated using the Linq-Embed-Mistral model [17], which has demonstrated strong performance on a number of benchmarks [16]. Dimensionality reduction was performed with UMAP (n_neighbors=6, n_components=10), followed by clustering with HDBSCAN (min_cluster_size=5). A fixed random seed (5991) was set to ensure reproducibility. Outputs included topic probabilities, 15 top keywords per topic, and up to 10 representative paragraphs per topic. Notably, all paragraphs were successfully assigned to a topic and no outliers were identified. Topics were then manually labeled based on their top keywords and representative paragraphs. Each topic was reviewed to ensure coherence and internal consistency.

## Results

**Figure 1:**
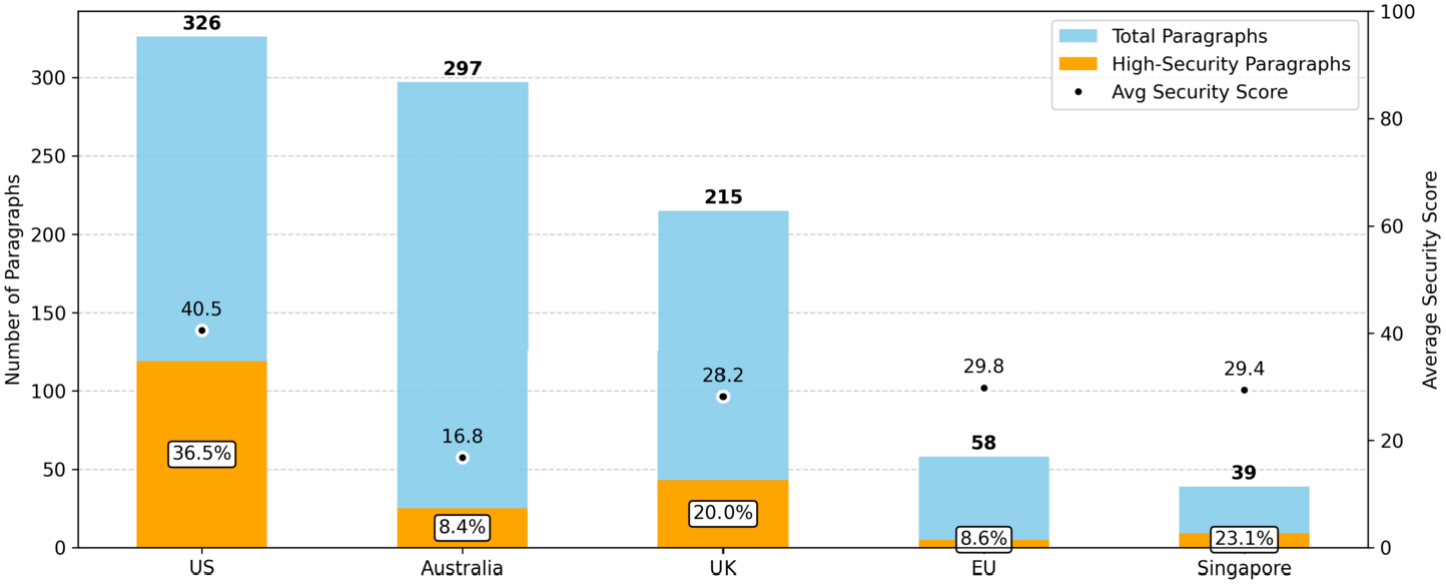
Parliamentary mentions of quantum technologies and security emphasis, 2010–2024. Bars show total paragraph counts per country, orange segments indicate high-security emphasis (score > 66). Black dots represent average security scores (right axis)

Figure 1 provides a bird’s-eye view of how often lawmakers in each legislature discuss quantum technologies and how strongly they link it to security concerns. Three observations stand out. First, the U.S. leads both in volume and in intensity. Roughly one-third of all 935 paragraphs in the corpus stem from the U.S. Congress (n=326). 36.5% of these clear the high-security threshold (> 66 points) and the mean security score of 40.5 is the highest across the sample. U.S. legislators not only talk about quantum technologies more frequently than their peers elsewhere, they also frame it as a security issue more often.

Second, Australian MPs talk frequently about quantum technologies yet rarely in security-laden terms. Only 8.4% of Australian contributions fall into the high-security category and the mean security score (16.8) is the lowest in the sample. Of the 297 Australian paragraphs, 76 (roughly 26%) relate to the government’s AUD 940 million deal with the Silicon Valley firm PsiQuantum. The debate about this investment is highly politicized; opposition MPs allege that the expression-of-interest process was engineered to benefit PsiQuantum at the expense of domestic firms, label the decision a “captain’s pick,” point to lobbying links, and have asked the Auditor-General to investigate. These exchanges center on procurement mechanics and industrial policy rather than defense or cybersecurity, which may, at least in part, displace security-related concerns and explain Australia’s comparatively muted security focus.

Third, the remaining regions fall somewhere in between. The UK shows a mid-range pattern, with 215 paragraphs and 20% classified as highly security-focused and a mean security score of 28.2. Singapore and the EU, by contrast, contribute far fewer total interventions, 39 and 58 paragraphs, respectively, but still exhibit mean security scores near 30. This suggests a comparatively moderate level of security framing even in the context of limited attention to quantum technologies generally. In summary, the securitization of quantum technologies is uneven. It is most pronounced in Washington, moderately salient in London, and muted in Canberra. In Brussels and Singapore, quantum technologies receive little legislative attention overall. When it is discussed, heavy emphasis on security is more prevalent in Singapore than in the EU.

### Temporal patterns

**Figure 2:**
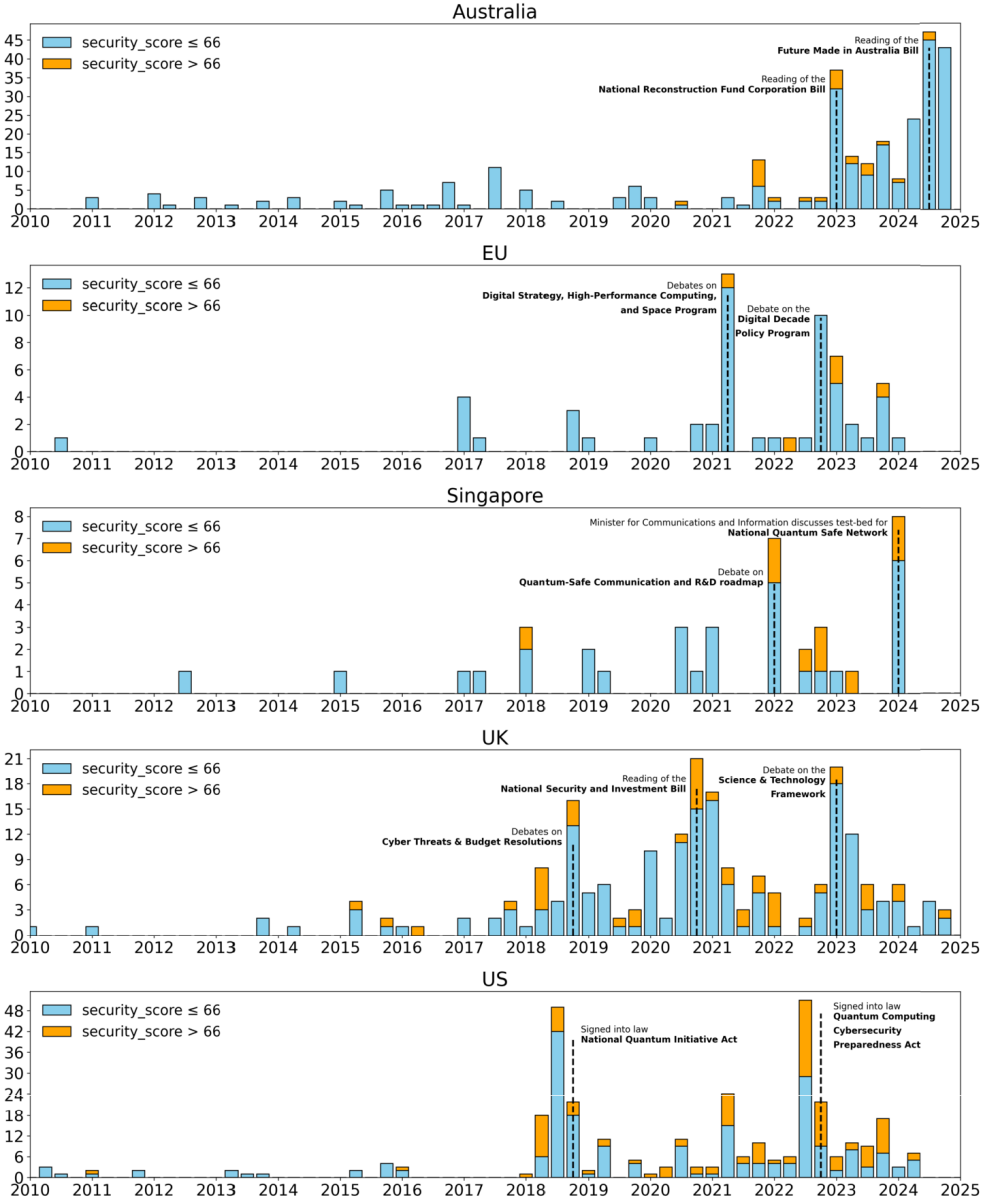
Quarterly paragraph counts by region. The blue bars represent paragraphs with low-to-moderate security emphasis and the orange bars indicate high-security emphasis. Each subplot uses a different y-axis scale to account for differences in volume between regions

Figure 2 illustrates quarterly trends in parliamentary discussions in all five legislatures from 2010 through late 2024. The blue segments represent paragraphs with low-to-moderate security emphasis, while the orange segments highlight those with a high security emphasis. As the figure shows, parliamentary attention to quantum technologies was minimal between 2010 and 2017. This sparse early activity contrasts with the period from around 2018 onward, when quantum technologies began to appear more regularly on legislative agendas, particularly in the UK and the US, and more recently, since 2023, in Australia, indicating a more sustained policy interest in quantum technologies.

In recent years of heightened activity, parliamentary debate on quantum technologies has tended to be sporadic rather than steady, with spikes often coinciding with high-profile legislative actions. In the US, for instance, peaks have coincided with the passage of legislation such as the *National Quantum Initiative Act* in late 2018 and the *Quantum Computing Cybersecurity Preparedness Act* in late 2022. Similarly, in Australia between 2023 and 2024, substantial attention to quantum technologies developed over debates about the *National Reconstruction Fund Corporation Bill*, a flagship industrial policy of the Albanese government, and the *Future Made in Australia Bill*, during which the PsiQuantum controversy was vigorously discussed.

The UK, EU, and Singapore exhibit similar episodic patterns in parliamentary engagement with quantum technologies, though the UK demonstrates a consistently higher baseline frequency compared to the EU and Singapore. These patterns are typically driven by targeted legislative debates, such as the discussions about the UK’s *National Security and Investment Bill*, the EU’s *Digital Decade Policy Program*, and Singapore’s *National Quantum Safe Network*. Outside these events parliamentary discussion remains limited. Thus, while quantum technologies periodically attract legislative attention, the frequency of debate remains largely episodic.

**Figure 3:**
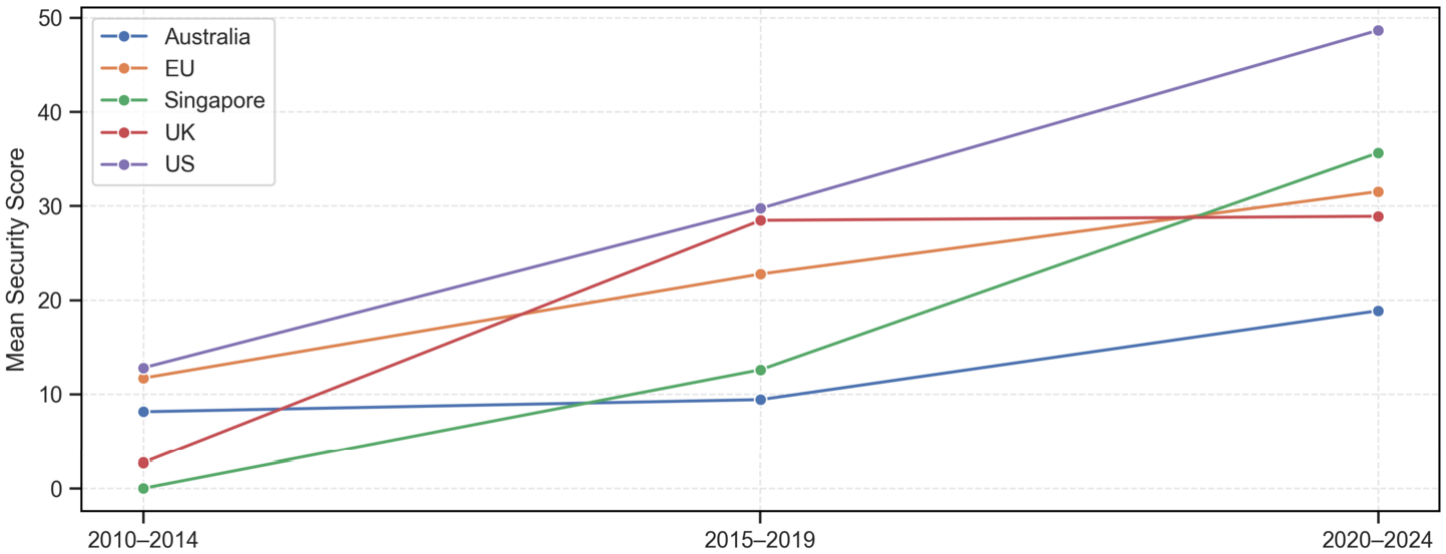
Security-emphasis scores for parliamentary speeches on quantum technologies, averaged by country over three five-year periods: 2010–2014, 2015–2019, and 2020–2024

In contrast, we do find an upward trend in security emphasis over time (see Fig. 3), albeit at different speeds across parliaments. The United States show the most consistent rise from one period to the next. In the UK, security rhetoric climbs sharply at first and then levels off in the most recent period. Speakers in the Australian legislature remain comparatively restrained, although there is an uptick in the period from 2020 to 2024. Security emphasis in Singapore and the EU also trends upward. Overall, the plot indicates a general increase in security language in debates about quantum technologies, most pronounced in the United States.

### Sentiment analysis

**Figure 4:**
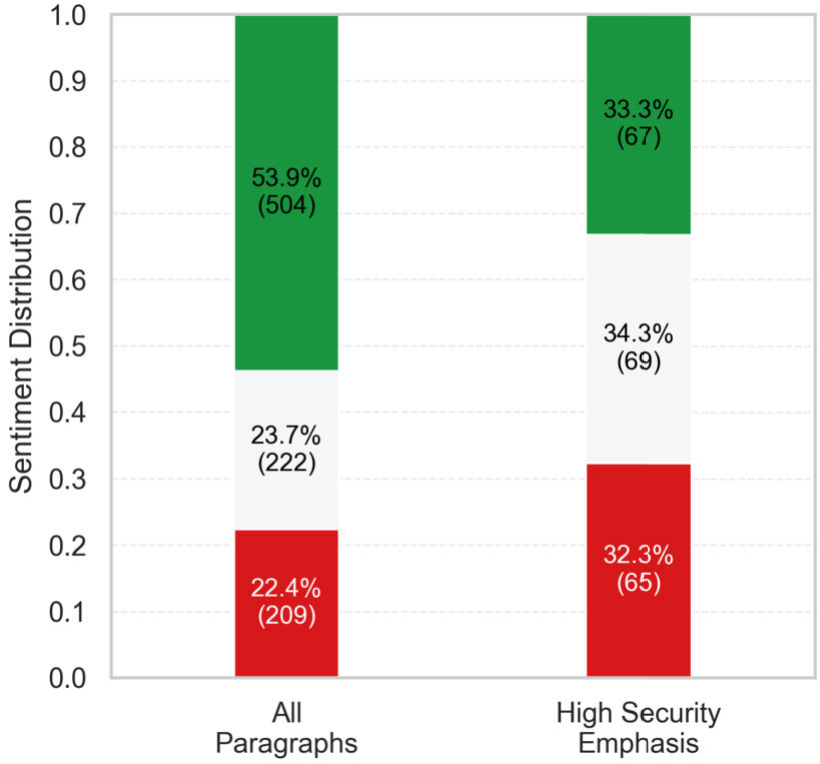
Sentiment distribution in all paragraphs vs. high-security paragraphs. Overall sentiment skews strongly positive (green), but shifts toward more neutral (grey) and negative (red) sentiment in paragraphs with a high emphasis on security

To examine how sentiment relates to security emphasis, we present two visualizations. The stacked bar chart in Fig. 4 shows that sentiment in the full corpus skews positive: 53.9% of all paragraphs are classified as positive, compared with 23.7% neutral and 22.4% negative. When we zoom in on paragraphs with high security emphasis (security score > 66), the distribution shifts markedly: the share of positive messages falls to 33.3%, while neutral and negative rise to 34.3% and 32.3%, respectively.

**Figure 5:**
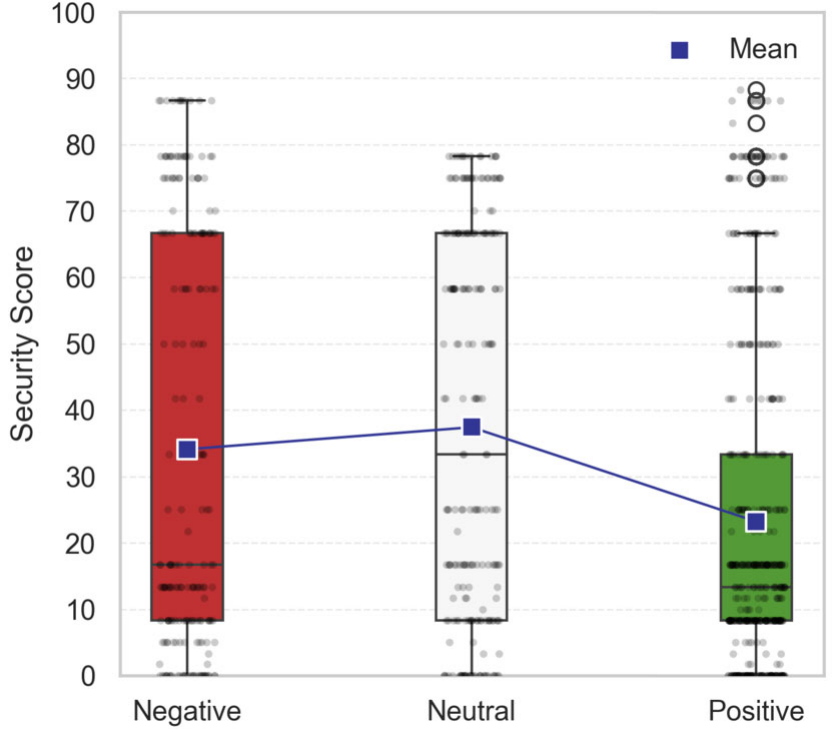
Boxplots of security scores by sentiment category. Security scores are, on average, higher for neutral and negative sentiments compared to positive sentiment and show greater variability as well

**Figure 6:**
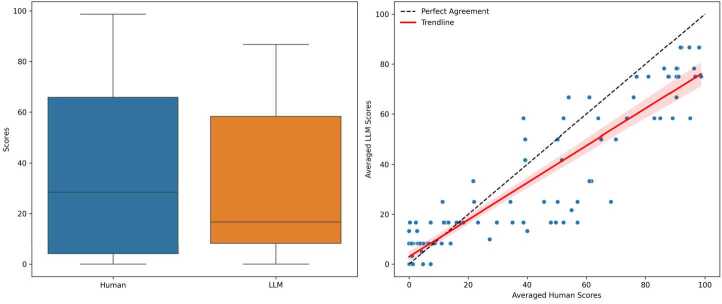
Comparison of human and LLM security scores. **Left:** Boxplot comparison shows the distribution of scores across human and LLM ratings. **Right:** The scatterplot shows the average LLM and human scores for 100 paragraphs, with a red trendline and a black line representing perfect agreement

Figure 5 corroborates this pattern. The boxplot displays the distribution of security scores across sentiment categories. Paragraphs with neutral and negative sentiment (mean = 37.4 and mean = 34.1, respectively) have higher average security scores than positive ones (mean = 23.2) and also exhibit a broader spread, reflected in larger inter-quartile ranges and higher upper whiskers. Taken together, the two visualizations show a systematic relationship. As security emphasis intensifies, the emotional tone of parliamentary debate becomes more variable and shifts from positivity toward greater caution, criticism, or disapproval.

This pattern aligns with securitization theory’s predictions about discourse transformation. According to Buzan et al. [4, 4], securitization represents the shifting of issues out of normal political bargaining processes into emergency mode. Our sentiment analysis empirically captures this shift. While general discussions of quantum technologies feature predominantly positive framing (53.9% overall), this optimistic tone gives way to more cautious and critical language as security emphasis intensifies (only 33.3% positive in high-security contexts). This shift from opportunity-focused to more threat-focused rhetoric is a form of “dramatization” which, as Buzan et al. [4, 26] argue, is used to justify “extraordinary means.” Ultimately, these results provide quantitative evidence of the emotional dimensions of the rhetorical transformation that securitization theory describes.

As previously mentioned (see Data & Methods), we ran a pre-trained RoBERTa sentiment classifier [22] on the same paragraphs as a robustness check. While its outputs did not exactly replicate the percentage distributions of our majority-vote LLM approach, they reproduced the same overall interpretative pattern: the corpus leans positive overall, while neutral and negative sentiment are more prominent in highly securitized speeches. Visualizations are provided in Appendix B.

### Topic modeling outcomes

The topic modeling analysis generated five general topics. Table 1 provides an overview of each, including representative keywords, paragraph counts, and sentiment distributions. We describe each topic in more detail below.

**Table 1:** Overview of BERTopic-Derived Topics in High-Security Emphasis Paragraphs

#	Label	Top Keywords	N	Sentiment (%)
0	Post-Quantum Cryptography and Infrastructure Protection	security, information, technology, data, encryption, federal, communications, national, cybersecurity, secure	73	Neg: 30 **Neu: 49** Pos: 21
1	Great Power Competition and R&D Leadership	china, artificial intelligence, technologies, national, research, technology, chinese, security, defense, ai	63	Neg: 40 Neu: 19 **Pos: 41**
2	AUKUS Partnership and Defense Tech	aukus, defense, capabilities, australia, security, government, intelligence, undersea, artificial intelligence, cybersecurity, nuclear	39	Neg: 18 Neu: 23 **Pos: 59**
3	Inbound Investment Screening and Merger Controls	security, national security, technologies, investment, mergers, dual use, computing hardware, instrument, hardware	18	Neg: 28 **Neu: 61** Pos: 11
4	Outbound Capital Restrictions and Financial Decoupling	american, chinese, companies, china, semiconductors, investment, investors, american investors, chinese companies, military	8	**Neg: 75** Neu: 12.5 Pos: 12.5

*Note: Sentiment values represent percentages based on the LLM majority-vote classification. Dominant sentiment values are shown in bold.*

#### Post-quantum cryptography and infrastructure protection

This topic focuses on cryptographic vulnerabilities and the transition to quantum-resistant digital infrastructures. Much of the data centers on the U.S. legislative response to the threat that quantum computers pose to current encryption systems. Agencies such as the Office of Management and Budget, the National Institute of Standards and Technology, and the U.S. Army’s Quantum Information Science Program are singled out for playing lead roles in coordinating the migration of government systems to post-quantum cryptography standards. Speakers identify multiple threat actors, particularly highlighting China’s cyberespionage capabilities and Russia’s demonstrated willingness to exploit infrastructure vulnerabilities, as evidenced by the 2020 hack of U.S. federal agencies.^9^

In several references to the U.K. Parliament, concerns about quantum threats to encryption are expressed, with officials from the intelligence agency GCHQ quoted to lend urgency to these concerns. Similarly, speeches from the European Union emphasize the vulnerability of critical communications infrastructure and advocate for coordinated, EU-wide adoption of post-quantum cryptographic standards to protect government and commercial networks. References to the Parliament of Singapore emphasize the central role of national agencies, such as the Cyber Security Agency, in developing quantum-secure communications infrastructures and protecting critical information systems.

#### Great power competition and R&D leadership

This topic addresses the race for technological supremacy across multiple frontier domains, with quantum computing positioned as one critical capability among several. Primarily voiced by U.S. Congress members, these speeches characterize China as a “full-spectrum peer competitor” investing over $14 trillion in quantum computing, robotics, biotechnology, and 5G networks. U.S. policymakers view these efforts as part of a broader geopolitical push to displace the U.S. as the global leader in economic and military power.

In their remarks, U.S. lawmakers emphasize the need to invest in foundational technologies, especially quantum computing, via defense appropriations, research funding, and industrial policy. They frequently highlight supply chain vulnerabilities, particularly the risks of relying on Southeast Asia for semiconductor manufacturing, and the dangers of foreign control over critical components used in advanced defense systems. Allied collaboration, especially with Europe and NATO, is framed as essential to accelerating dual-use innovation, sustaining U.S. democratic leadership. Collectively, the speeches frame quantum technologies as a potential source of both military and economic power and as an important domain in the competition with authoritarian regimes.

#### AUKUS partnership and defense technology

AUKUS is portrayed as a two-pillar security partnership that includes the long-term effort to build an Australian fleet of conventionally-armed, nuclear-powered submarines (Pillar 1) and an effort to co-develop high-tech capabilities in domains such as cyber-defense, AI, hypersonic missiles, electronic warfare, and quantum science (Pillar 2). Legislators and ministers (mostly from Australia, but also from the United Kingdom and the United States) describe quantum technologies as essential to preserving technological advantage in secure communications, sensing and navigation, and data analytics in the Indo-Pacific.

They also frame quantum technologies as both an industrial opportunity and a means of achieving collective security through allied collaboration. Research budgets, national reconstruction funds, and talent pipelines must be synchronized among the three partners to ensure development of hardware and cryptographic capabilities within the alliance. While submarines may not reach the water for years, multiple speakers stress that encryption tools and navigation aids can be fielded much sooner. Similarly, joint strategies on semiconductors and quantum technologies are promised to harmonize supply-chain security and R&D to ensure that the bloc remains at the “leading edge” of defense technology.

#### Inbound investment screening and merger controls

The topic concentrates on the UK government’s effort to strengthen security safeguards against corporate takeovers and technology exports. Members of parliament mention a draft statutory instrument that will lower merger-control thresholds so that more deals in three sensitive areas can be scrutinized: military and dual-use goods, computing hardware (including semiconductors), and quantum technologies. These measures complement the National Security and Investment Act 2021, a UK law that grants the government the power to scrutinize and intervene in business acquisitions, and reflect wider sanctions that already restrict exports of high-tech and dual-use items to states such as Belarus and Russia.

Running through the debate is a concern about hostile access, particularly by China, to UK know-how. Speakers highlight the scale of Chinese students at UK universities, the dominance of firms like Huawei, and the risk of technology “leakage” or industrial espionage through corporate acquisitions and research partnerships. They emphasize that quantum technologies, alongside semiconductors and AI, represent strategic assets where foreign ownership or control could compromise national security. The consensus is that tightening merger scrutiny and export controls is a proportionate regulatory response to prevent sensitive capabilities from falling into adversarial hands.

#### Outbound capital restrictions and financial decoupling

This topic addresses concerns about outbound capital flows that inadvertently strengthen adversaries. Speakers warn that American pension funds, hedge funds, and private-equity firms are pouring capital into Chinese companies that build semiconductor chips, AI, quantum computers, or hypersonic missiles, technologies seen as decisive for 21st-century warfare by American politicians. Because Beijing’s “military-civil fusion” doctrine blurs any line between civilian and military use, that capital is said to boost China’s armed forces and could one day threaten U.S. troops in a conflict over Taiwan.

To blunt that risk, the speakers press for (1) mandatory disclosure of where U.S. money goes, (2) legal limits on or screening of investments tied to sensitive technologies, and (3) greater domestic investment and in military alliances. The overall aim, lawmakers assert, is targeted financial decoupling, not a blanket ban on all business with China.

## Discussion

We examined *how often* (RQ1) and *in what ways* (RQ2) elected officials cast quantum technologies as matters of security. Across 935 parliamentary paragraphs (2010–2024), three patterns emerged. First, securitizing rhetoric is unevenly distributed, it is most intense in the United States, moderate in the United Kingdom, and muted in Australia. Parliamentary discussions of quantum technologies in the European Union and Singapore are noticeably more limited in volume than in other countries. Second, every legislature exhibits an upward trend in security emphasis over time. Third, although a majority of speakers express positive sentiment about quantum technologies, they adopt a more negative tone when addressing security implications.

Country-specific narratives vary but share recurring motifs. U.S. debates foreground great-power competition with China and call for export controls, alliance coordination, and higher defense spending. Australian debates focus more on industrial policy than security, particularly the contested PsiQuantum investment, and UK discourse emphasizes the screening of foreign investment and merger control. EU MPs stress technological sovereignty and “de-risking,” while Singaporean legislators emphasize critical-infrastructure protection and quantum-secure communications. In addition, two cross-cutting dynamics stand out: (1) engagement is concentrated in the AUKUS member states (Australia, the United Kingdom, the United States), which account for most references; and (2) industrial-policy and security discourse are often tightly coupled. Debates on sovereign capability, supply-chain resilience, and large investment vehicles routinely segue into arguments about regulating capital investment, alliance readiness, and great-power rivalry.

### Policy implications

Securitizing quantum technologies poses a “Goldilocks dilemma” for policymakers, who must determine the optimal approach. Under-securitization risks complacency and irresponsibility; migration to quantum-proof cryptography in critical sectors may be delayed and unchecked foreign investment may enable malign actors. However, an excessive focus on security measures may impede the collaborative efforts that have been essential for scientific progress. The risk of expansive restrictions is not merely hypothetical. Our analysis shows that parliamentary debates feature calls for restrictions on financial and equipment flows (see topics no. 3 and 4). Recent export controls on quantum technologies illustrate these risks. Multiple jurisdictions have imposed restrictions that extend beyond hardware to encompass knowledge exchange and international research collaborations, with some measures criticized by the scientific community as excessively broad or lacking technical justification [26]. In line with others [33], we therefore argue that rather than pursuing restrictions that stifle collaboration, policymakers should aim for targeted regulation that protects the collaborative frameworks and open data sharing essential to advancing a field where many core technologies are not yet mature and still require fundamental research.

### Implications for securitization theory

Our analysis makes three contributions to securitization theory. First, we advance the methodological toolkit by developing and validating an LLM-based text-scaling approach that measures security emphasis at scale. Furthermore, while scholars have theoretically linked emotions to securitization processes [11, 32] and demonstrated that computational methods are well suited to illuminate securitization dynamics [35], we explicitly connect an empirical measure of sentiment to securitization theory. We thereby provide evidence for the rhetorical transformation that the theory implies. That is, as security emphasis intensifies, discourse shifts from predominantly positive to more negative and critical language.

Second, we offer, to our knowledge, one of the first quantitative, cross-national, longitudinal analyses of parliamentary discourse on quantum technologies. We show not only that securitization is increasing, but also how it varies in volume, timing, and thematic focus across political systems. Capturing elite-level deliberation reveals how elected representatives frame quantum technologies for constituents and fellow lawmakers. Patterns suggest that approaches to technology governance and strategic posture (e.g., defense commitments, alliance participation, position in geopolitical rivalries) shape securitization, though not deterministically. The United States and United Kingdom, with permissionless innovation models and AUKUS-based cooperation, exhibit high to moderate security emphasis; the EU’s values-based regulatory approach and Singapore’s distinct posture outside Western alliances correspond to more moderate securitization and overall lower engagement with quantum technologies. Australia’s comparatively muted security framing despite AUKUS membership indicates that domestic policy controversies mediate broader structural drivers.

Third, quantum technologies satisfy all three conditions that Buzan et al. [4] identify as facilitating securitization: (1) the grammar of security; (2) the authority of securitizing actors; and (3) recognizable threat features. For (1), topic modeling shows classic securitizing moves that invoke existential risks (breaking encryption, adversaries achieving military advantage) and advocate extraordinary measures (export controls, financial screening, accelerated cryptographic migration), drawing on established security vocabularies of espionage, military supremacy, and infrastructure protection. For (2), speeches are delivered by legislators, cabinet ministers, and defense officials who possess elevated social capital speaking from institutional platforms with formal authority over national-security policy; they frequently cite expert bodies (e.g., GCHQ), layering institutional authority and expert knowledge to strengthen securitizing claims. For (3), quantum technologies are clearly dual-use, with recognizable threats (cryptographic vulnerabilities, espionage capabilities, military sensing advantages, and supply-chain dependencies). In addition, the epistemic opacity of quantum technologies, stemming from the abstract and hard-to-grasp nature of quantum mechanics and the novelty of quantum applications, limits the ability of lay audiences to verify claims about potential threats, likely reduces contestation, and increases deference to expert opinion. We therefore add to securitization theory by proposing that, for high-tech emerging technologies, epistemic opacity itself functions as a feature that facilitates securitization.

### Limitations & future research

This study has some limitations that point to future work. First, it omits speeches from executive-branch and agency officials. This is particularly salient for the European Union, where the Commission, holding the EU’s near-monopoly right to initiate legislation, may employ stronger security framing than Parliament. Unlike national parliaments where legislative initiative is formally shared between parliament and government, the European Parliament is a co-legislator with the Council but lacks a general right to initiate legislation; it amends and adopts measures largely on Commission proposals. Our findings reflect parliamentary discourse and do not capture Commission rhetoric. Second, the dataset is weighted toward Western parliaments. Extending the sample to non-Anglophone democracies (e.g., South Korea), emerging economies (e.g., Brazil), and non-democratic systems (e.g., China) would sharpen cross-cultural comparisons. Third, we focus exclusively on quantum technologies and do not compare their securitization to other emerging technologies (e.g., artificial intelligence, biotechnology, nanotechnology). Within-country comparisons across multiple technologies would clarify whether the patterns we observe are domain-specific or part of general legislative responses to emerging technologies.

## Figures and Tables

**Figure 7:**
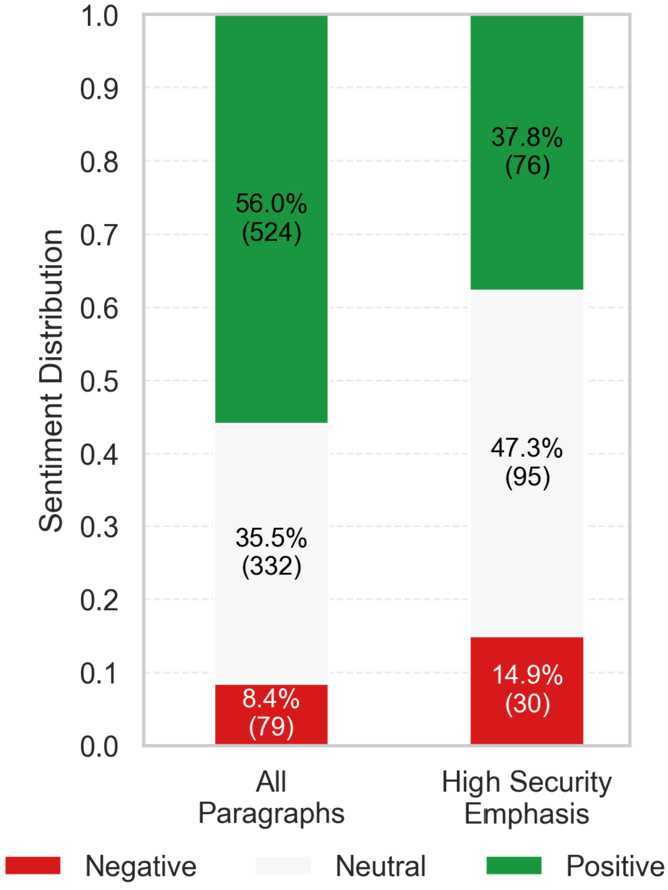
RoBERTa sentiment distribution for all paragraphs and the high-security subset

**Figure 8:**
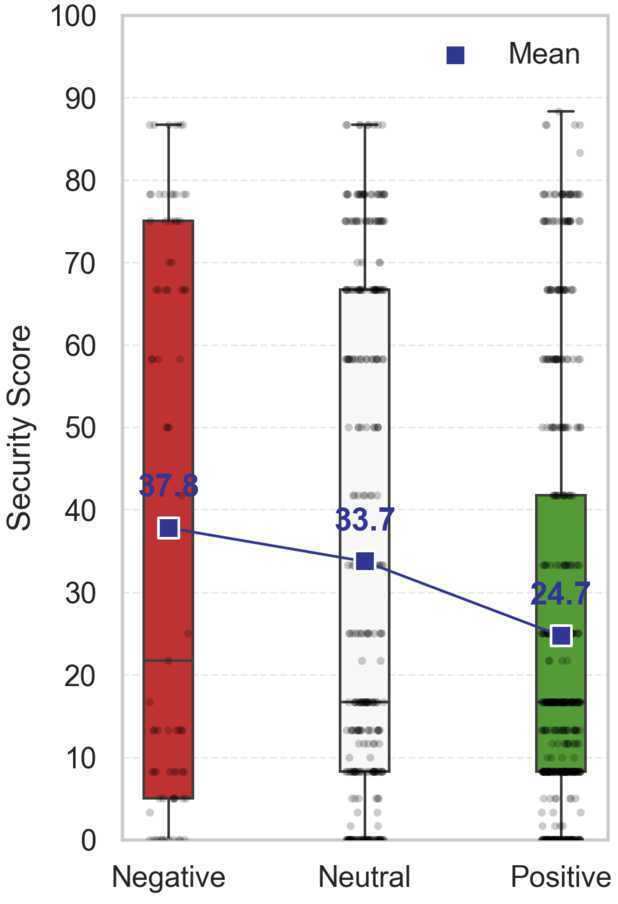
Security score box-plots by sentiment category using RoBERTa labels

## Data Availability

The dataset supporting this study has been uploaded to Zenodo and is accessible via the following DOI: https://doi.org/10.5281/zenodo.15938130

## Appendix A: Reliability metrics

**Table 2:** Inter-coder reliability among human raters for 100-paragraph sample. Pairwise agreement is measured using Pearson correlation

Metric	Estimate
Coder 1 – Coder 2	0.833
Coder 1 – Coder 3	0.722
Coder 2 – Coder 3	0.817
ICC(3,1)	0.812
Krippendorff’s *α*	0.804

**Table 3:** Inter-model reliability among LLMs for 100-paragraph sample. Pairwise agreement is measured using Pearson correlation

Metric	Estimate
Sonnet – Mistral	0.817
Sonnet – GPT-4o	0.841
Mistral – GPT-4o	0.815
ICC(3,1)	0.840
Krippendorff’s *α*	0.794

**Table 4:** Agreement between averaged human and averaged LLM scores for the 100-paragraph sample

Metric	Value
Pearson Correlation	0.918
Intraclass Correlation (ICC[3,1])	0.898
Krippendorff’s *α*	0.877

## Appendix B: Sentiment analysis robustness check

As a robustness check, we compared our sentiment analysis results with those produced by a pre-trained RoBERTa classifier. Figure 7 shows that the RoBERTa labels mirror our findings. The full corpus leans positive, while neutral and negative sentiment become more prominent in high-security passages. Figure 8 confirms that neutral and negative paragraphs have higher and more variable security scores than positive ones. This convergence across approaches strengthens confidence in the findings.

## Footnotes

1 Navigation systems that function without relying on Global Navigation Satellite Systems like GPS or Galileo.

2 Light detection and ranging systems that use quantum properties like entanglement to enhance sensitivity and accuracy in target detection and range estimation.

3 https://www.aph.gov.au/Parliamentary_Business/Hansard.

4 https://www.europarl.europa.eu/plenary/en/debates-video.html.

5 https://sprs.parl.gov.sg/search/#/home.

6 https://hansard.parliament.uk/.

7 https://www.congress.gov/congressional-record.

8 Temperature is a parameter that controls the randomness of LLM outputs. A temperature of 0 produces deterministic, highly consistent responses, while higher values introduce more variability in the model’s responses.

9 See https://www.cisa.gov/news-events/cybersecurity-advisories/aa20-352a and https://en.wikipedia.org/wiki/2020_United_States_federal_government_data_breach.
